# Acute hepatitis E virus infection as a trigger for postinfectious IgA nephropathy: A rare case report

**DOI:** 10.1097/MD.0000000000046341

**Published:** 2025-12-26

**Authors:** Musawer Khan, Zarafshan Khan, Ashfaq Altaf, Safia Bibi, Ishrat Batool, Abed El Rahman Naamani, Nour Fakih

**Affiliations:** a Department of Nephrology, Combined Military Hospital Quetta, Quetta, Pakistan; b Quetta Institute of Medical Sciences, Quetta, Pakistan; c Gilbert and Rose-Marie Chagoury School of Medicine, Lebanese American University, Byblos, Lebanon; d Department of Natural Sciences, Lebanese American University, Beirut, Lebanon.

**Keywords:** acute kidney injury, hepatitis E, IgA nephropathy, renal biopsy, viral infections

## Abstract

**Rationale:**

IgA nephropathy (IgAN) is most often idiopathic but may occur following infections. While associations with hepatitis B and C are recognized, its link with acute hepatitis E virus () infection is rarely described and remains poorly understood.

**Patient concerns:**

A 37-year-old male presented with fever, sore throat, vomiting, generalized weakness, and dark-colored urine for 2 days.

**Diagnoses:**

Laboratory evaluation showed markedly elevated liver enzymes, hyperbilirubinemia, and acute kidney injury. Hepatitis E IgM was positive. Renal biopsy demonstrated postinfectious IgA nephropathy with mesangial hypercellularity and moderate acute tubular injury.

**Interventions:**

The patient received supportive therapy, including intravenous fluids, hemodialysis, plasma exchange, and corticosteroid treatment with intravenous methylprednisolone followed by an oral prednisolone taper.

**Outcomes:**

Renal function progressively improved. By 3 months, serum creatinine decreased from 703 µmol/L at admission to 135 µmol/L, with resolution of symptoms and no recurrence of hematuria or renal impairment.

**Lessons:**

This case suggests that acute HEV infection may act as a trigger for postinfectious IgA nephropathy. Clinicians should consider IgAN in patients with HEV who develop renal dysfunction, as timely recognition and management can substantially improve outcomes.

## 1. Introduction

IgA nephropathy (IgAN) is characterized by recurrent hematuria and IgA deposition in the glomerular mesangium, often exacerbated by upper respiratory tract infections.^[1]^ It is characterized by mesangial IgA deposits, leading to glomerulonephritis and potential kidney dysfunction.^[2]^ While most cases of IgA nephropathy are idiopathic, secondary causes can include viral infections, chronic liver disease, and other systemic conditions.^[3]^ The association between hepatitis E infection and IgA nephropathy is not widely reported, making this case of particular interest. Hepatitis E is a common viral infection that typically resolves spontaneously in immunocompetent individuals but can lead to more severe manifestations in immunocompromised hosts.^[4,5]^ The purpose of this case report is to highlight the rare connection between hepatitis E virus (HEV) and IgA nephropathy, which has not been extensively documented in the medical literature.

## 2. Case presentation

### 2.1. Patient information

A 37-year-old male from Quetta, Pakistan, was admitted to the emergency room on December 7, 2024, with complaints of fever, sore throat, generalized body weakness, multiple episodes of vomiting, and dark-colored urine for 2 days. He had no prior comorbidities or surgical history. Family history was significant for hypertension and diabetes mellitus in his father, but no hereditary renal disease was noted. He had recently traveled to Lahore but had no known sick contacts.

Upon admission:

*Vital signs*: BP 125/78 mm Hg, pulse 91 bpm, RR 20 breaths/min, temperature 98°F.

*Physical exam*: Bilateral pedal edema and scrotal swelling, with no pallor, cyanosis, or jaundice.

*Respiratory exam*: Coarse crackles and decreased air entry in bilateral lung fields.

*Abdomen*: Mild tenderness in the right hypochondrium, with no organomegaly.

### 2.2. Timeline

**Table:** 

Event	Date
Symptom onset	December 5, 2024
Hospital admission	December 7, 2024
Initiation of hemodialysis	December 12, 2024
Renal biopsy	December 20, 2024
Initiation of plasma exchange	December 22, 2024
Initiation of methylprednisolone	December 25, 2024
Discharged from dialysis	January 28, 2025
Follow-up at 3 months	April 28, 2025

### 2.3. Preoperative laboratory and imaging findings

**Table:** 

Laboratory test	Result
Total leukocyte count	10.8 × 10^9^/L
Hemoglobin	12.8 g/dL
Platelets	134 × 10^9^/L
Serum ALT	6318 U/L
Serum albumin	38 g/L
Total bilirubin	57 µmol/L
Serum creatinine	703 µmol/L
24 h Urine protein	1202 mg
RBCs in urine	8–14/HPF
Serum CRP	50 mg/L
INR	4.72
Hepatitis A, B, C	Negative
Hepatitis E IgM	Positive

**Figure 1.**
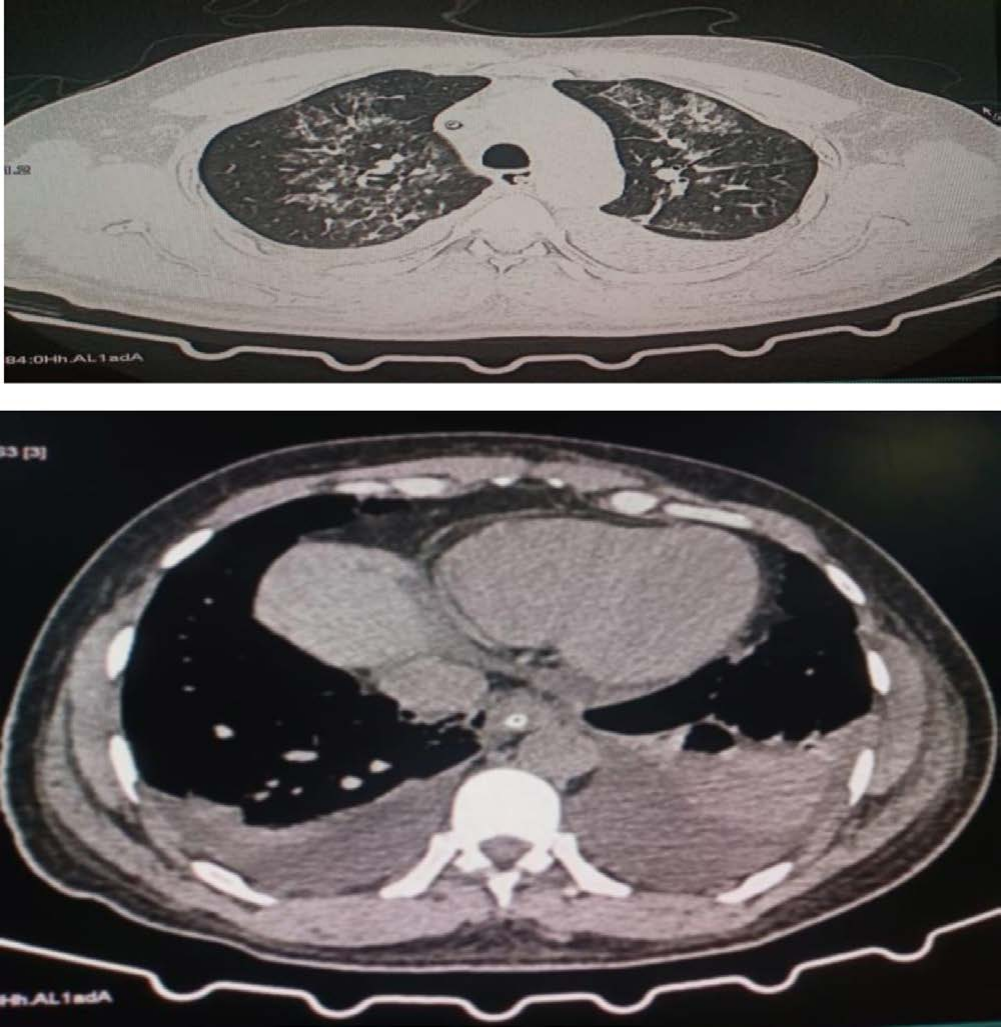
High-resolution CT scan showing bilateral mild pleural effusions and features consistent with pulmonary edema.

Ultrasonography showed increased renal parenchymal echogenicity. High-resolution CT revealed bilateral mild pleural effusions and pulmonary edema (Fig. 1). Additionally, the scan demonstrated bilateral mild pleural effusions with bibasilar and right middle lobe consolidations. Smooth septal thickening was observed in the bilateral lower lobes, middle lobe, and lingula, along with ground-glass opacities, with findings suggestive of pulmonary edema or fluid overload. Mild cardiomegaly with a streak of pericardial effusion was also noted.

### 2.4. Diagnostic assessment

**Figure 2.**
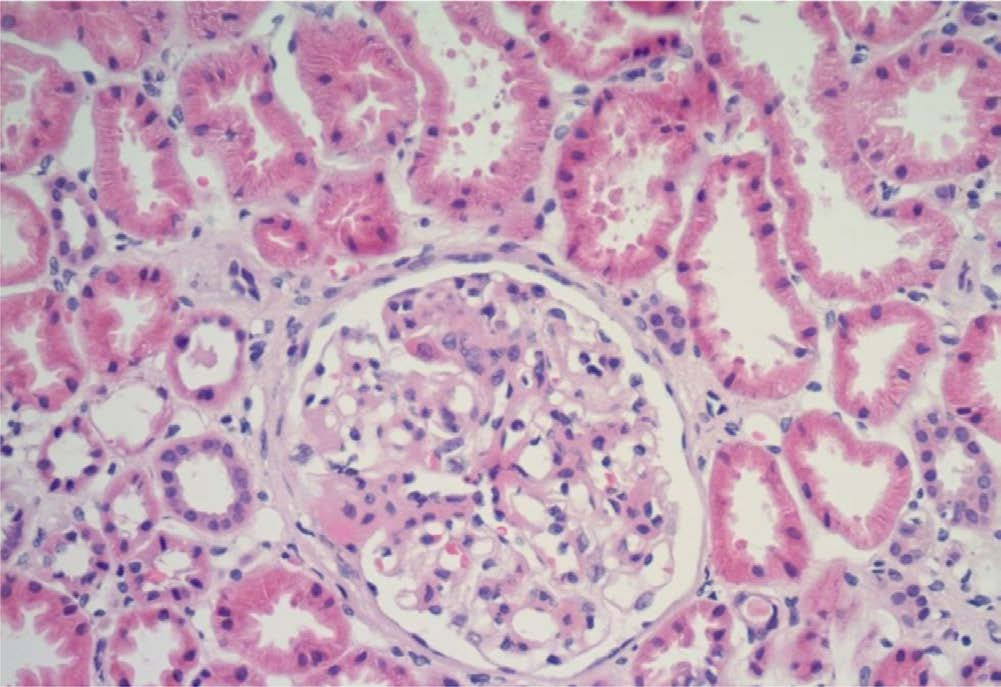
Histopathological examination of the renal biopsy demonstrating mesangial hypercellularity and tubular necrosis.

**Figure 3.**
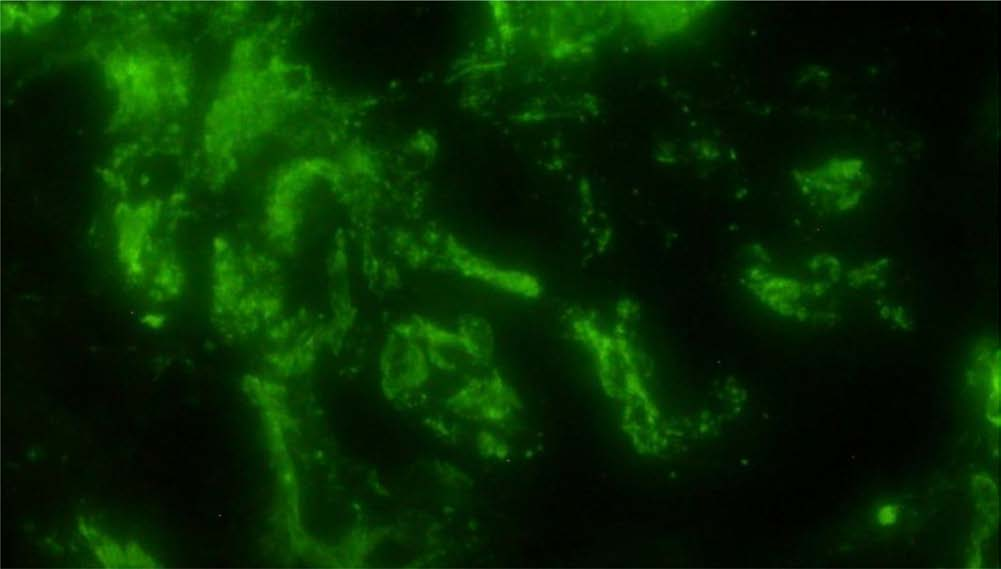
Immunofluorescence analysis of the renal biopsy sample revealing IgA deposits in the mesangium, characteristic of IgA nephropathy.

A renal biopsy confirmed postinfectious IgA nephropathy with focal mesangial hypercellularity and moderate acute tubular necrosis (Fig. 2). Immunofluorescence demonstrated moderate IgA deposits without evidence of interstitial fibrosis or crescent formation (Fig. 3).

### 2.5. Differential diagnosis

Acute viral hepatitis (HEV): Confirmed by positive hepatitis E IgM and clinical presentation.

Acute kidney injury: Present with glomerular features (hematuria and proteinuria), findings inconsistent with hepatorenal syndrome. Prerenal causes were excluded based on history, examination, and investigations for dehydration, blood loss, gastroenteritis, myocardial infarction, and renal artery stenosis. Post-renal and other renal causes were ruled out through urine microscopy, ultrasonography of kidneys and prostate, CT of the kidneys, ureters, and bladder, and renal biopsy.

Postinfectious glomerulonephritis: Confirmed by renal biopsy findings demonstrating mesangial hypercellularity and IgA deposition.

Acute tubular necrosis: Although moderate acute tubular injury was observed on renal biopsy, it was considered a secondary process and not the primary cause of renal dysfunction, as the dominant finding was mesangial IgA deposition consistent with postinfectious IgA nephropathy.

### 2.6. Therapeutic intervention

The patient received intravenous fluids, antiemetics, and pain management. Hemodialysis was started on day 5, and plasma exchange therapy (5 sessions over 10 days) was initiated on day 15. After renal biopsy confirmed IgA nephropathy, treatment was initiated with intravenous methylprednisolone 1 g daily for 3 days, followed by oral prednisolone 1 mg/kg/d in 2 divided doses.

This regimen was selected based on institutional experience for postinfectious glomerulonephritis with severe renal dysfunction, balancing efficacy with safety. A gradual taper of oral steroids was continued for 3 months.

### 2.7. Follow-up and outcomes

At follow-up, renal function improved significantly, with a reduction in proteinuria and stabilization of the glomerular filtration rate. By 3 months post-discharge, serum creatinine had decreased to 135 µmol/L and serum urea to 10.5 mmol/L. The patient remained asymptomatic, with no recurrence of hematuria or renal impairment.

## 3. Discussion

HEV is a leading cause of acute viral hepatitis worldwide.^[6]^ It is estimated that 1 in 8 individuals has been infected with HEV at some point in their lifetime.^[7]^ The high prevalence of HEV can be attributed to its transmission primarily through the fecal-oral route, particularly via contaminated water sources,^[8]^ which has contributed to its higher incidence in developing countries and areas with inadequate sanitation.^[9]^

While HEV infection is usually self-limiting in immunocompetent individuals, it can lead to more severe complications, particularly in immunocompromised hosts. Numerous systemic manifestations of HEV have been documented, with the most notable being neurological symptoms such as Guillain–Barré syndrome, neuralgic amyotrophy, and encephalitis. Additionally, renal manifestations such as membranoproliferative and membranous glomerulonephritis have been reported.^[10]^ However, to our knowledge, postinfectious IgA nephropathy (IgAN) as a complication of HEV infection has been described only once in the literature.^[11]^ This highlights the importance of considering IgAN as a differential diagnosis when renal involvement is observed in patients with acute HEV, especially since management and prognosis vary significantly among different types of glomerulonephritis.

The patient in this case initially presented with fever, sore throat, generalized body weakness, vomiting, and dark-colored urine. Initial laboratory tests indicated an ongoing infectious process. Upon further investigation, a markedly elevated serum ALT of 6318 U/L and proteinuria of 1202 mg in 24 hours suggested both hepatologic and renal involvement. Hepatitis serologies were performed, and only hepatitis E IgM returned positive, confirming a recent HEV infection and ruling out chronic or concomitant infections with hepatitis A, B, or C. HEV RNA polymerase chain reaction testing was not available at our institution at the time of presentation. Therefore, the diagnosis of acute hepatitis E was based on compatible clinical features and positive HEV IgM serology, in accordance with World Health Organization diagnostic criteria. This approach has been validated in resource-limited settings where polymerase chain reaction facilities are unavailable. Further evaluation of the renal pathology through a renal biopsy revealed IgA nephropathy, as confirmed by immunofluorescence staining.

Although a renal biopsy is a relatively invasive procedure compared to urinalysis, it remains the gold standard for confirming the diagnosis and ruling out other kidney diseases, such as acute tubular necrosis.^[12]^ Acute kidney injury was present with glomerular features (hematuria and proteinuria), with findings inconsistent with hepatorenal syndrome.^[13]^ This further supported the diagnosis of IgA nephropathy, which was confirmed by biopsy findings.

Since the patient was not immunocompromised, the hepatitis E infection was self-limiting and was managed with supportive care. This highlights the critical role of serological testing in identifying the causative agent. For instance, while hepatitis B and C require antiviral treatment,^[14,15]^ the management of hepatitis E focuses on supportive care. Regarding the nephropathy, before the final diagnosis of IgA nephropathy was established, the patient was initiated on hemodialysis, as his serum creatinine levels were rising significantly after hospitalization. Hemodialysis was preferred over peritoneal dialysis due to the acute increase in serum creatinine. If the disease had transitioned into chronic kidney disease, peritoneal dialysis would have been considered, as it has been shown to offer a better quality of life for patients compared to hemodialysis.^[16]^ Treatment was initiated with intravenous methylprednisolone 1 g daily for 3 days, followed by oral prednisolone 1 mg/kg/d in 2 divided doses. This regimen was selected based on institutional experience for postinfectious glomerulonephritis with severe renal dysfunction, balancing efficacy with safety.

Although the precise mechanisms linking hepatitis E to IgA nephropathy remain unclear, our case report suggests that HEV may trigger the development of IgAN. This finding is important for monitoring patients diagnosed with HEV for potential kidney injury. Furthermore, it underscores the need for clinicians to consider a recent history of HEV infection when evaluating patients with IgA nephropathy and no clear inciting event. In regions endemic for HEV, physicians should maintain a high index of suspicion for this combination of renal and hepatic dysfunction in such patients.

Emerging research offers valuable insights into the immunopathogenesis underlying this renal involvement. A study from the University of Zurich examined kidney tissue from HEV-infected immunocompromised patients and identified glomerular deposition of the HEV ORF2 capsid protein, a key viral antigen.^[17]^ Notably, this protein was found in the absence of detectable HEV RNA in the kidney tissue, indicating an immune-mediated rather than a direct cytopathic mechanism of injury. Specifically, a truncated and non-glycosylated variant of the ORF2 protein was shown to form immune complexes with circulating immunoglobulins, resulting in glomerular inflammation and injury. These findings support the hypothesis that HEV may act as an immunologic trigger for glomerular diseases, including IgA nephropathy. This mechanistic insight complements our case and reinforces the need for clinicians to maintain a high index of suspicion for postinfectious glomerulonephritis in HEV-infected patients presenting with renal dysfunction.

However, the findings from our report are limited by the fact that it describes a single case. Therefore, conclusions regarding causality between HEV and IgA nephropathy should be made cautiously. Further case series and prospective studies are necessary to validate this potential association and explore the molecular and immunologic pathways involved. Long-term follow-up of patients is also essential to confirm viral clearance and monitor for recurrence or progression of renal disease.

## 4. Conclusion

This case report highlights the rare but significant association between HEV infection and IgA nephropathy. While HEV is typically a self-limiting illness, it can lead to unexpected complications, including renal manifestations such as postinfectious IgA nephropathy. Early recognition of this connection is crucial for clinicians, particularly in areas where HEV is endemic, as it can guide appropriate treatment strategies and improve patient outcomes. Although the mechanisms linking HEV to IgA nephropathy remain unclear, this case adds valuable insight into the potential renal complications of HEV infection. Further research and larger cohort studies are needed to explore the molecular basis of this relationship and establish a definitive causal link. In the meantime, clinicians should maintain a high index of suspicion for IgA nephropathy in patients with HEV infection who present with renal dysfunction. Early diagnosis, timely intervention, and close monitoring are essential to prevent further renal damage and ensure the best possible outcomes for affected patients.

## Author contributions

**Investigation:** Zarafshan Khan, Ashfaq Altaf, Safia Bibi, Ishrat Batool.

**Methodology:** Zarafshan Khan, Ashfaq Altaf, Safia Bibi, Ishrat Batool.

**Project administration:** Musawer Khan.

**Supervision:** Nour Fakih.

**Writing – original draft:** Musawer Khan, Abed El Rahman Naamani, Nour Fakih.

**Writing – review & editing:** Abed El Rahman Naamani, Nour Fakih.
